# 25(OH) vitamin D serum values and rheumatoid arthritis disease activity (DA S28 ESR)

**Published:** 2014

**Authors:** Maryam Sahebari, Zahra Mirfeizi, Zahra Rezaieyazdi, Houshang Rafatpanah, Ladan Goshyeshi

**Affiliations:** 1 Rheumatic Diseases Research Center, School of Medicine, Mashhad University of Medical Sceinces, Mashhad, Iran

**Keywords:** 25(OH)D, vitamin D, Rheumatoid Arthritis, RA, DAS 28 (ESR).

## Abstract

***Background: ***The role of vitamin D in the pathogenesis of rheumatoid arthritis is under investigation. This study was designed to evaluate the correlation between serum values of 25(OH) vitamin D [25(OH)D] and disease activity in rheumatoid arthritis (RA) patients according to Disease Activity Score 28 joints and ESR (DA S28 ESR).

***Methods:*** Ninety-nine patients according to ACR classification criteria for RA and 68 healthy controls were included in this study. The participants with known confounding risk factors affecting serum values of 25(OH)D were excluded. All patients were under treatment with supplementary calcium carbonate (1500mg), 25(OH)D (800U), and Hydroxychloroquine (6mg/kg). The control group was mostly recruited from patients’ relatives who lived with them to minimize the impact of diverse lifestyles on 25(OH)D status. Disease activity was assessed by DA S28 ESR. Serum concentrations of 25(OH)D were measured. Serum values of 25(OH)D less than 50 nmol/L were considered 25(OH)D deficiency.

***Results:*** The mean 25(OH)D serum values were 83.74±46.45 nmol/L in patients and 46.53±34.07 nmol/L in controls. After adjustment for age, sex and BMI, multivariate analysis showed no correlation between 25(OH)D serum levels and DAS in RA (P=0.29, r_p_=0.11). However, 25(OH)D serum values were significantly lower in patients with early diagnosed RA compared with the other patients (p=0.012). In the early diagnosed patients, 25(OH)D and anti-CCP serum values were negatively correlated (P=0.04, r_s_=-0.5).

***Conclusion: ***This study showed that there was no correlation between 25(OH)D serum values and DAS over a short duration of disease course. However, in early RA, 25(OH) D serum values were lower than the established RA.

Rheumatoid arthritis is a chronic autoimmune disease characterized by loss of self-tolerance leading to immune activation particularly against synovial tissues (1). Its pathogenesis is still under investigation, although the role of genetic susceptibility, environmental factors, and immune activation is indispensable (1). Nowadays, there are cumulative data about the modulation of innate and adaptive immune cells by 25(OH)D, and extensive evidence of dysregulation of T cell subsets in 1, 25(OH)D related pathways for initiation and perpetuation of inflammatory arthritis (2-6). Although several studies strongly suggest, if not proven, that 25(OH)D therapy attenuates autoimmune disorders, epidemiological studies still have not shown whether 25(OH)D deficiency is a primary phenomenon or an outcome of autoimmune diseases (2-10). 

Besides, long-term follow-up of some epidemiological studies have revealed that 25(OH)D deficient healthy individuals do not show any increment of RA risk  (11). In addition, the immunomodulatory serving dose of 25(OH)D is not exactly defined yet (12). The key question which now arises is whether the serum values of 25(OH)D are reliable indicators of disease activity in autoimmune diseases with fluctuating courses like RA or not. A few studies investigated the correlation between RA disease activity and vitamin D serum levels (10-11, 13-18). Controversies in the aforementioned study results may stem from several factors affecting 25(OH)D values. So, we decided to evaluate the correlation between the serum values of 25(OH)D, the best indicator of extra renal production of 1,25(OH)D, and disease activity in RA patients. 

## Methods

Ninety-nine patients diagnosed with RA according to the ACR criteria and sixty-eight healthy controls were included in this study (19). The study was conducted in Mashhad, Iran, located at 36.20º latitude and 59.35º east longitude. It should be mentioned that Iran is a country with four distinct seasons. The New Year in the country falls in April. During the first six months -April to September- the weather tends to be sunny and warm, and indeed hot. In the second half of the year-October to March- it is usually cold and cloudy. Physical examination, medical history and blood biochemistry were evaluated in all patients to exclude systemic diseases such as chronic kidney or liver disease, malnutrition, and malabsorption (serum albumin below 2.2 mg/dl, cholesterol below 100mg/dl and BMI <18.5kg/m^2^). Participants with BMI≥30kg/m^2^ were also excluded (regarding the hypothesis which states that 25(OH)D is fat soluble and lower 25(OH)D in overweight individuals may simply be representative of a larger volume of distribution of this vitamin) (20). Moreover, patients with a history of chronic diarrhea, gastrectomy, patients who received supra physiologic doses of vitamin D (>800U/day) and anti convulsive therapy during a 6-month period prior to the study were omitted from the study, too. All of the patients in this study were treated with supplementary calcium carbonate 1500 mg and 25(OH)D (maximum dose:800U/ day) and hydroxychloroquine 6mg/kg in addition to glucocorticoids and methotrexate. Since we can not stop vitamin D intake or change our patients’ treatment regimen to make a homogenous group we selected patients with the same medications which are suspected to affect vitamin D serum levels. The control group was mostly recruited from the patients’ acquaintances that lived with them (patient’s mother or sisters) to minimize the influence of lifestyle, dressing and nutritional habits on 25(OH)D status in both the case and control groups. The control group was matched for age, gender, and race with the patient group. This control group including normal volunteers was selected to compare the serum values of Vit D in RA patients all according to age and sex matched group. 

The blood samples were taken from brachial vein in the morning, and then they were separated by centrifugal method, stored and refrigerated minus 20 degrees. 25(OH) Vitamin D was measured by Elisa. (Immundiagnostik AG, Bensheim, Germany). Anti-CCP antibody serum values more than 5 units were considered elevated according to the manufacturer’s instruction (Euroimmune, Germany). Vitamin D status was defined as follows: serum values equal or more than 75nmol/L, 50nmol/L≤25(OH)D< 75nmol/L, and less than 50nmol/L were considered 25(OH)D sufficiency, insufficiency and deficiency, respectively (21-24).

Disease activity was assessed in accordance with the Disease Activity Score including 28 joint counts (DA S28 ESR). Components of DAS28 are ESR, patient-assessed visual analogue scale (VAS) (0–100), and swollen and tender joint counts (both from 0–28). Highly active disease was defined as DAS28 > 5.1; moderately active disease 3.2 < DAS28 ≤ 5.1; and slightly active disease DAS28 ≤ 3.2.

All participants signed an informed consent and the local Ethical Committee approved the protocol. The SPSS Version 11.5 was used for the statistical analysis. Kolmogorov-Smirnov test was used to evaluate the normality of data. Values were expressed as mean±SD for normally distributed variables and median with inter-quartile range (IQR) for non-normally distributed data. Baseline demographics and clinical characteristics were compared between groups using independent samples *t*-test, Mann-Whitney U test, chi-square, One-Way ANOVA and/or Fisher’s exact test when appropriate. Bivariate correlations were assessed using Pearson’s and Spearman’s correlation coefficients for normally and not-normally distributed data, respectively. Multivariable linear regression analysis was used to determine the interaction between 25(OH)D and DAS. Potential confounding variables were evaluated including age, sex, season of serum sampling and drug history. Stepwise procedures determined the significant variables to remain in the model. 

## Results

In this study, 167 (99 RA patients and 68 healthy volunteers) participated. The mean age of the total participants was 42.37±14.07 years. The mean age of patients and controls was 43.94±14.31 and 39.87±13.41 years respectively with no statistical difference, (P=0.074). We did not find any statistical difference in sex distribution among the case and control groups (P=0.09) (table 1).

The mean of disease duration was 5.9±5.6 years (0.2-20 years). The mean of DAS28ESR was 4.45±1.67. The mean serum values of creatinine, alkaline phosphatase, calcium, phosphorus, albumin and cholesterol, were within normal limits (table 2). The median IQR of anti-CCP in these patients was 20(5-78) units (normal range less than 5 units). 75.7% of patients showed the serum levels of 25(OH)D≥ 50 nmol/L while 32.3% of the healthy volunteers had 25(OH)D serum values equal to or more than 50 nmol/L. With regard to age, the patients were divided into three groups: younger than 40 years, 40 to 60 years and older than 60 years. 25(OH)D in these groups was studied showing that there was not any statistical difference among these groups (P=0.12) (table 1).

The mean serum value of 25(OH)D in total participants in this study was 68.31±45.53 nmol/L. Based on the general linear model controlling for age (P=0.004, r=0.22) and gender (P=0.53,r=0.05), 25(OH)D was significantly higher in patients compared with controls (P<0.001, r=-0.4). 

The patients were divided into two groups according to seasons of sampling. In patients with autumn and winter onset the 25(OH)D serum levels were 75.44±45.63 nmol/L; in other patients, 25(OH)D serum levels were 85.63±46.79 nmol/L. There was not any statistical difference between these two groups (p=0.38). 25(OH)D (nmol/l) serum values in RA patients according to DAS28ESR were as follows, A: DAS≤2.5 (66.99±39.7), B: 2.5<DAS≤3.2 (63.35±28.8),C: 3.2<DAS≤5.1 (80.46±49.7), D: DAS>5.2 (94.11±45.1). (P=0.17). The results of Tukey-test for comparing the above groups in pair were not significant (p>0.05). Considering 25(OH)D serum values as dependent factor (VitD≤ 35 & VitD> 35 nmol/l) and possible confounders including sex, age, and BMI, disease duration and season of sampling and DAS28ESR, the logistic regression analysis showed none of the aforementioned factors especially DAS associated with 25(OH)D. Season of sampling (the first six spring and summer months) (P=1, β(SE)= -19.9±40192.9), Sex (female) (P=0.8, β(SE)=0.2±1.2), disease duration (P=0.4,β(SE)=-0.4±0.06), BMI (P=0.2, β(SE)=-0.08±0.07), age (P=0.1, β(SE)=0.04±0.02) and DAS 28 ESR (P=0.7, β(SE)=0.5±0.1). 

**Table 1:** Comparison of the number (percentage) of patients and controls in three groups regarding the 25(OH)D serum values and the distribution of serum values of 25(OH)D according to age classification in patients and controls

**Variables**		**RA Patients n(%)**			**Healthy controls n(%)**		
Serum value Of VitD (nmol/L)	Age group	69.96±23.75	76.62±42.9	73.46±43.65	45.5±39.05	50.94±30.21	44.41±21.0
	A:<40(n=80)						
	B:40≥age<60 (n=67)	102.84±61.6	93.29±43.05	94.85±49.80	42.5±24.21	46.61±27.44	41.61±14.21
	C:≥60(n=20 )	62.60±11.1	83.36±48.89	81.2±46.9	82.02±24.52	89.02±21.98	68.01±31.12
P value (One-Way ANOVA)*		0.12			0.059		
Serum value of Vit D (nmol/L)		90.21±52.03	83.09±46.2	83.74±46.45	46.53±34.07	60.58±31.43	42.89±34.0
p value (t-test)		0.63		<0.001 (case & control group)		0.08	

Tukey test:

P value between A& B groups, in patients: 0.08; in controls: 0.94

P value between A&C groups, in patients: 0.87; in controls: 0.04

P value between B&C groups in patients: 0.68; in controls: 0.03

**Table 2:** Important laboratory & demographic data of Rheumatoid arthritis patients

**Laboratory & demographic Parameters**	**Mean±SD** **or** **Median(IQR)**	**Correlation with VitD serum values**
Calcium (mg/dl)	9.26±0.56	P=0.85,r=0.02
Phosphorous (mg/dl)	3.8±0.58	P=0.43, r=0.09
Alkaline Phosphatase(Unit)	145(115-180)	P=0.71, r= -0.06
Cholesterol(mg/dl)	216±110	P=0.56, r=-0.07
ESR (mm/hr)	27.9±23	P=0.74, r= 0.03
Albumin (g/dl)	4.4± 0.63	P=0.2, r=-0.13
Rheumatic factor (RF) (U/ml)	40(13-67)	P=0.7, r=0.04
Hemoglobin (gr/L)	12.3±1.8	P=0.81,r=0.02
Platelet (n/mm^3^)	303x10^3^±14x10^3^	P=0.59, r=0.06
White blood cell count(n/ mm3)	8.2x10^3^±6.2x10^3^	P=0.14,r=-1.6
Disease Duration (years)	5.5±5.2	P= 0.9, r=-0.013
Age (years)	42.37±14	P=0.004, r=0.2
BMI (Kg/m^2^)	26.8±4	P=0.037, r=-0.26
Visual analogue scale (VAS) (unit)	47.5±27.7	P=0.2, r=0.1
Tender joints (n)	4.4±4.8	P= 0.3, r=0.14
Swollen joints(n)	8.8±8.5	P=0.8, r=0.02
Methotrexate Dosage (mg/week)	8.3±5.4	P=0.96,r=-0.005
Prednisolone Dosage (mg/d)	5.9±3.7	P=0.4,r=0.8

Pearson or Spearman correlation test as appropriate

**Figure 1:**
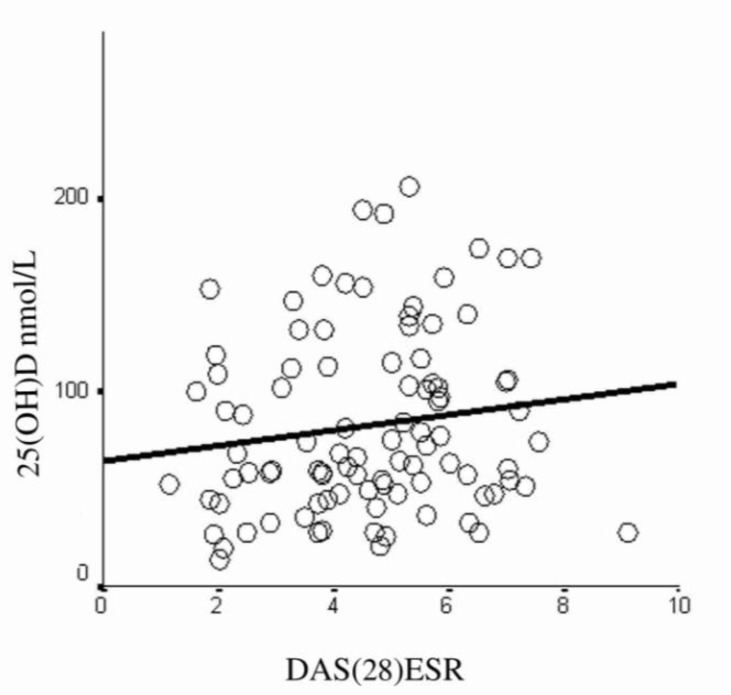
Vitamin D and disease activity, no significant correlation was found between these two parameters (p=0.29, r=0.11)

**Figure 2:**
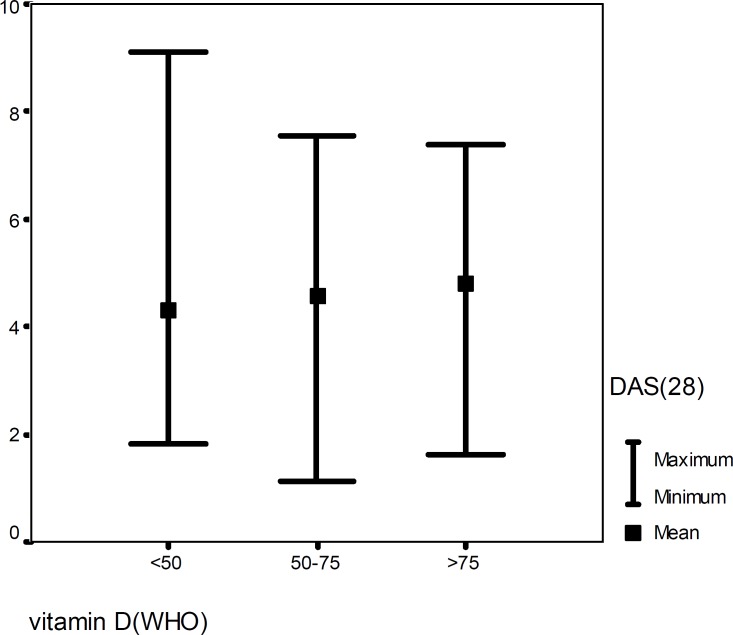
Distribution of DAS28ESR in different serum values of 25(OH)D, p=0.51 (One-Way ANOVA)

Figure 1 illustrates that there was no correlation between 25(OH)D serum values and DA S28 ESR (P=0.29,r_p_=0.11). In addition, we found no correlation between duration of disease, anti CCP, ESR, VAS, and the number of tender and swollen joints with 25(OH)D serum concentrations (table 2). According to the results projected in table 2, there was no correlation between 25(OH)D serum values and drug history. When DAS28ESR was compared among the three categories of 25(OH)D serum values, there was not any difference in mean values of DAS among them (P=0.51) (figure 2). 

Applying the DA S28 ESR categorization criteria to four groups, 83.3% of patients were diagnosed with the active disease (DAS 28 ESR>2.6). 25(OH)D serum levels were significantly lower in the new cases with disease duration of less than 6 months (22 patients) compared with the chronic patients (P=0.012). In these new cases, anti-CCP and 25(OH)D serum values showed a negative correlation with 25(OH)D, (P=0.04,r_s_=-0.5).

25(OH)D serum values< 50nmol/L: Mean of DAS: 4.3±1.83

50nmol/L≤ 25(OH)D serum values≤75 nmol/L: Mean of DAS: 4.55±1.69

25(OH)D serum values> 75nmol/L: Mean of DAS: 4.79±1.57 

## Discussion

The most obvious finding to emerge from this study is that 25(OH)D serum concentrations in RA patients under treatment with physiologic doses of 25(OH)D do not correlate with disease activity over a short period of time. Moreover, when the patients were divided into different groups according to DAS scores, serum concentrations of 25(OH)D did not show any statistical difference. In addition, in different serum values of 25(OH)D, given the accepted immunomodulatory serum levels of 25(OH)D, the disease activity of RA patients did not demonstrate any difference. Furthermore, we found that 75.7% of patients had 25(OH)D serum levels more than 50 nmol/L, while 83.3% of these patients had high disease activity. Another important result of this study was that, 25(OH)D serum values were lower in the newly diagnosed RA patients. In addition, in these patients, 25(OH)D and anti-CCP serum levels were negatively correlated. However, these results may be biased because of the longer duration of calcium carbonate (1500 mg) and 25(OH)D (800U) therapy in chronic patients. 

Although several studies have been conducted on the 25(OH)D serum values in RA patients compared with healthy controls, there are limited studies about the correlation between 25(OH)D and disease activity in these patients (table 3) (8, 11). As noted the documents in agreement with the regulatory role of 25(OH)D on the specific facets of human immunity have been shown to be on the rise in recent years and it seems that ,in the future, correction of vitamin D deficiency will be a part of immunomodulation strategies in several autoimmune diseases (5-6, 8, 11-13, 25). Nevertheless, the association of serum values of 25(OH)D as a surrogate of 1,25(OH)D body supply, with disease activity is not supported by most of the limited studies conducted with this purpose (2, 26-29). It is now confirmed that 1,25(OH)D is the most effective metabolite of 25(OH)D at the cellular level of immune cells (2, 28, 30). However, the direct influence of 25(OH)D on some of innate immune cells should not be ignored. The results of our study are in line with most of the previous studies on the correlation between DAS and 25(OH)D serum levels. There are some explanations for these findings. First, in this study, the patients were under treatment with physiological doses of 25(OH)D according to the recommendations for the osteoporosis prevention (800U/d). Although the patients under treatment with larger doses of 25(OH)D, for at least in the last six months before the study, were excluded, the mean serum values of 25(OH)D were at optimal and probably immunomodulatory levels (75-100nmol/L) (12, 31). It is now not clear though, in which serum values 25(OH)D plays an immunomodulatory role. However, it is proposed that serum values more than 100 nmol/L may result in pathologic fractures (12, 29). Therefore, the average serum values of 25(OH)D in our patients, being treated with physiological doses of 25(OH)D, have been recently the accepted range for Vit D sufficiency. Of course, we should not ignore the fact that prescribing hydroxychloroquine as one of the main DMARDs in all of our patients assist in keeping serum values of active metabolites of 25(OH)D at optimal levels (27). Therefore, it may be concluded that when the serum concentrations of 25(OH)D are sufficient, higher levels of 25(OH)D may not attenuate disease activity. Given the new treatment strategies, some researchers have investigated the clinical efficacy of therapeutic doses of 25(OH)D in the induction of remission of RA that was not effective (21, 32, 33).

**Table 3:** Summery of the studies conducted on correlation between DAS and VitD serum values in RA patients

**Name/year of the study**	**Number of patients**	**Age** **(year)**	**VitD serum values** **(mean±SD)** **(ng/ml)**	**Correlation be** **tween DAS & VitD** **(Yes/No)**	**Country**	**Inclusion & exclusion criteria**
Braun-Moscovici Y et al.(23) /2009	85	55.8±14.1	14.9±8.4(ng/ml)	No	America	50% received VitD (physiologic doses in 3previous months) 75% were treated with DMARDS
Kerr g et al. (21) / 2010	1181	64±11.3	27.7(ng/ml)	No	America	VitD & DMARD intake was controlled in 3 previous month,
Craig SM et al. (38)/2010	226	51.3±13.2	40.5±15 (nmol/l)	No	America	New cases(<2years) were included,18% received supplementary VitD
Turhanoğlu AD et al.(22) /2010	65	46±11.87	104.87±60.08(nmol/l)	Yes P=0.0001, r= -0.2	Turkey	Endocrine diseases were excluded
Cutolo M et al.(31) /2010	118	58.5±1.1 (Italy) 56.3±2.3 (Estonia)	Italy(winter) 58.9±5.4(nmol/l) Italy(summer) 65.2±5.4(nmol/l) Estonia (winter) 35.1±1.9(nmol/l) Estonia (summer) 46.4±2(nmol/l)	Yes P=0.0001, r=-0.57	Italy	Patients did not receive Vit D supplements, and were treated with prednisolone and DMARDs at least 3 months before the study
Current study /2011	99	43.94±14.3	83.74±46.4(nmol/l)	No	Iran/ Mashhad	All patients received 800U VitD and hydroxychloroquine before capturing. Patients with any other medical conditions except for RA, and known risk factors for VitD deficiency were excluded from the study likewise overweight patients. The influence of sampling seasons, in addition to BMI, age and sex was analyzed.

Second, most of the studies have proposed that 25(OH)D deficiency is a predisposing factor for the initiation and progression of autoimmune process. For instance, several studies have shown that 25(OH)D serum levels are lower in the newly diagnosed RA patients compared with healthy controls (2-3, 5, 7, 34). In the current study, we also found that 25(OH)D serum values are lower in early-diagnosed RA patients compared with those suffering from an established disease. Moreover, we observed a negative correlation between serum levels of 25(OH)D and anti-CCP in recently diagnosed patients. Kerr et al. also reported a relationship between low 25(OH) D body supplies and positive anti-CCP autoantibodies in RA patients. These findings may be in line with the hypothesis of 25(OH) D deficiency as a predisposing factor for the initiation of autoimmune process. Likewise, recently, it has been proposed that 25(OH)D supplementation may be more effective in prevention rather than treatment of infectious diseases like tuberculosis (21). Recent investigations have projected the influence of vitamin D receptor (VDR) genotypes and vitamin D binding proteins (BDPs) on responses to 25(OH)D supplementation. Researchers have also proposed that replacing new protocols for the measurement of 25(OH)D serum values to increase the precision of the assay will be able to help with better results in the determination of 25(OH)D status in patients (21, 33). Vitamin D deficiency in normal population of our study, especially in women, is unexpectedly high. As mentioned previously, healthy volunteers in this study in contrast to patients, did not receive 25(OH)D. However, the conservative dress code in the country, nutritional 25(OH)D deficiency, which is due to inaccessibility of 25(OH)D enriched food products in our country, and sedentary lifestyles are the main reasons for the high prevalence of 25(OH)D deficiency in our healthy population. Nevertheless, in this study, in which the control group was mostly selected from the patients’ relatives who lived with them, to decrease the effect of lifestyle on 25(OH)D status as a confounder, we observed that supplementary physiological doses of 25(OH)D can rise the serum values of 25(OH)D to the accepted normal range. The current study was not without limitations, though. This cross sectional study did not permit a power calculation on the causal relationship between 25(OH)D deficiency and RA activity. Moreover, there were several immeasurable confounders affecting 25(OH)D serum levels that could not be omitted from this study. The strongest point of our study was its precise inclusion and exclusion criteria, which are summarized in table 3.

In brief, in this study, we confirmed that the serum values of 25(OH)D are not a good indicator of disease activity. However, the newly diagnosed RA patients showed lower serum values of 25(OH)D compared with those with chronic disease. Another important outcome of the present study was that there is a negative correlation between serum values of 25(OH)D and anti-CCP in early RA. It should be mentioned that future studies considering new protocols of 25(OH)D measurements, the role of genetic variations and DBPs in 25(OH)D status are necessary to clarify the exact role of 25(OH)D in autoimmune diseases like RA. Furthermore, defining new end points for the clinical response to the 25(OH)D serum levels’ fluctuation is required to understand the impact of 25(OH)D supplementation on disease activity or progression. 

## Notes

**Funding: **The Research Council of Mashhad University of Medical Sciences, Mashhad, Iran financially supported this study with grant number [87835]. This research was extracted from Dr Ladan Ghoshayeshi’s thesis to fulfill the internal medicine specialty degree requirements.
